# 

**Published:** 1885-01

**Authors:** 


### CONTENTS.

### COMMUNICATIONS.

Protection of the Cervical Border........................................... 5
President's Address.......................................................... 8
Operative Dentistry ......................................................... 10
A Singular Case.............................................................. 11

### PROCEEDINGS.

Northwestern Dental Association ............................................. 13
Nineteenth Annual Meeting of the Ohio State Dental Society ......... 14
CORRESPONDENCE ............................................................ 37

### SELECTIONS.

Discussion of Miller's Theory at the last Meeting of the American Dental Society, of Europe ............................................... 40
Review of Miller's Theory of Dental Caries.................................. 43
Camphor Inhalations in Coryza................................................ 44
Cocaine ......................................................................... 46
Salicylic Acid as a Prophylactic Against Cholera........................... 47
Electric Surgery ............................................................... 47
Panacea for Trouble ........................................................... 48
Shortsightedness .............................................................. 48
SALMAGUNDI.................................................................... 49

### EDITORIAL.

The End of the Year ........................................................... 53
Listerine ....................................................................... 54
International Medical Congress of 1887...................................... 55
Floral Guide.................................................................... 56
The Physician's Visiting List for 1885 ...................................... 56

